# Surgical Treatment of Chronic Giant Left Ventricular Pseudoaneurysm

**DOI:** 10.1155/2021/4308690

**Published:** 2021-02-10

**Authors:** Roberto Ramos Barbosa, Assad Miguel Sassine, Vitor Martinelli Batista Rolim, Pietro Dall'Orto Lima, Eduardo Moreno Judice de Mattos Farina, Ramon Chiabai Moura, Luiz Fernando Machado Barbosa

**Affiliations:** ^1^Department of Cardiology, Santa Casa de Misericórdia de Vitória Hospital, Rua Dr. João dos Santos Neves, 143, Vila Rubim, 29025-023 Vitória, ES, Brazil; ^2^School of Medicine of Santa Casa de Misericórdia, Av. Nossa Sra. da Penha, 2190, Bela Vista, 29027-502 Vitória, ES, Brazil

## Abstract

Left ventricle pseudoaneurysm is usually a severe complication of acute myocardial infarction, caused by rupture of the myocardial wall with pericardium bleeding. Mortality can reach 50 to 80% within a week if not properly treated. Hemodynamic instability, cardiac tamponade, and cardiac arrest are life-threatening presentations that require surgical treatment. We report a case of a man with a left ventricle chronic giant pseudoaneurysm and unspecific symptoms. After critical judgement on a heart team basis, surgical treatment was successfully performed, with a good long-term clinical outcome.

## 1. Introduction

Left ventricular pseudoaneurysm (LVPA) is caused by rupture of the left ventricle wall leading to communication between the chamber and the pericardium. It is usually a life-threatening condition presenting after a complicated acute myocardial infarction (AMI). Mortality varies from 50 to 80% within the first week if not treated [1]. The incidence of LVPA is not well known, although it is lower than left ventricle free wall rupture, a critical mechanical AMI complication with a similar mechanism that occurs in 2 to 4% of all AMI cases [2, 3].

Hemodynamic instability with signs of cardiac tamponade is the most common clinical presentation of complicated LVPA, requiring a surgical approach in an emergent setting. In this scenario, the risk of cardiac arrest and death is imminent [4]. Other initial symptoms include pleuritic chest pain, agitation, and vomiting [5], with variable sensibility and specificity. LVPA in a non-AMI clinical context is a rare condition. However, different etiologies such as congenital, traumatic, postoperative, and inflammatory have been described for LVPA and left ventricular true aneurysm [6–8]. We report a challenging case of an elder patient with a giant LVPA accidentally diagnosed, undergoing successful surgical treatment.

## 2. Case Report

A 77-year-old man with progressive dyspnoea and fatigue at moderate exertion for the last two years was admitted to the Emergency Department on February 5, 2019, with nausea and abdominal pain. He felt the symptoms for three days and reported one episode of syncope after vomiting. He had been a smoker for 58 years and had no history of past AMI or chest pain. Electrocardiogram (ECG) showed a right bundle branch block, cardiac troponins were normal, and serum creatinine was 1.75 mg/dL. When interrogated, he affirmed that he had similar symptoms 45 days before, with nausea, vomiting, and abdominal discomfort followed by syncope. At that time, he was discharged from the Emergency Department after ECG and cardiac troponin dosages.

An abdominal ultrasound showed proximal left ureterolithiasis with moderate hydronephrosis. While planning urologic intervention, an abdominal computer tomography (CT) scan was performed and a cardiac enlargement was accidentally found, with mild left pleural effusion. A double-J ureteric catheter was placed, and a cardiac diagnostic workup was electively initiated.

**Figure 1:**
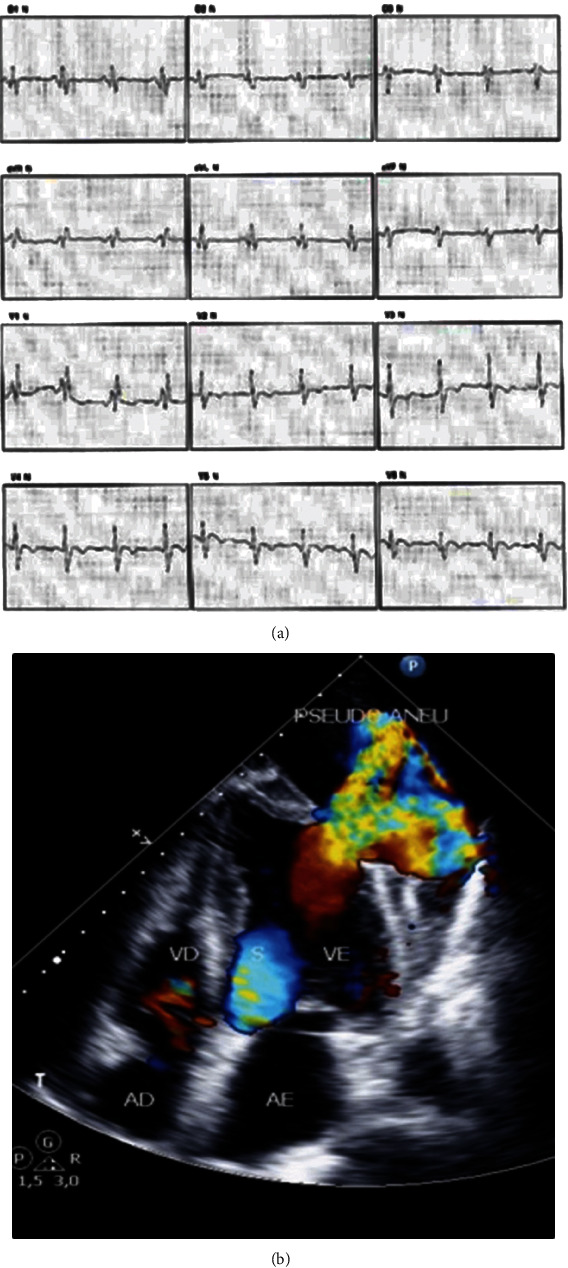
Resting electrocardiogram and transthoracic echocardiogram. (a) Resting electrocardiogram showing right bundle branch block, negative anterior wall T-wave, and lateral wall Q-wave. (b) Transthoracic echocardiogram showing left ventricular pseudoaneurysm and rupture of the apical segment of lateral myocardial wall of the left ventricle. pseudoaneu = pseudoaneurysm; VE = left ventricle; VD = right ventricle; AE = left atrium; AD = right atrium.

A treadmill exercise test showed a resting ECG with sinus rhythm, right bundle branch block, negative anterior wall T-wave, and lateral wall Q-wave (Figure 1(a)), and no ECG changes were found during exercise. Transthoracic echocardiogram showed rupture of the apical segment of the lateral myocardial wall of the left ventricle, with drainage from the cardiac chamber to an external cavity located on the lateral-inferior-apical region, with bidirectional flow suggesting an LVPA (Figure 1(b)). The ejection fraction via the Teichholz formula was 69%, and it was calculated with exclusion of the pseudoaneurysm.

**Figure 2:**
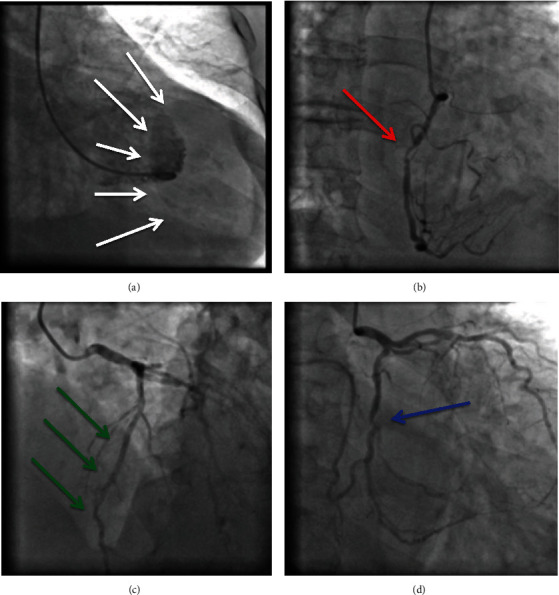
Left ventriculogram and coronary angiogram. (a) Left ventriculogram in right oblique view showing important contrast extravasation from the left ventricle to the pericardium forming a giant pseudoaneurysm (white arrows). (b) Coronary angiogram in right oblique view showing severe stenosis (80% lumen stenosis) in middle segment of the right coronary artery (red arrow). (c) Coronary angiogram in left cranial view showing normal left anterior descending artery (green arrows). (d) Coronary angiogram in right caudal view showing intermediate stenosis (40% lumen stenosis) in the left circumflex artery (blue arrow).

**Figure 3:**
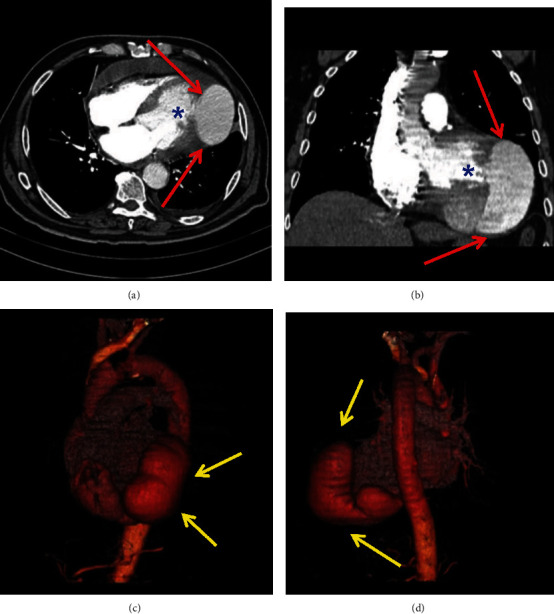
Chest computed angiotomography. (a, b) Large pericardial effusion (red arrows) and area of discontinuity in the posterolateral myocardial wall of the left ventricle communicating with the pericardial sac (blue asterisk). (c, d) Three-dimensional reconstruction by computed angiotomography revealing left ventricle giant pseudoaneurysm (yellow arrows).

The patient was referred for cardiac catheterization. Coronary angiogram revealed a normal left anterior descending artery (LAD), intermediate stenosis in the left circumflex artery (LCX) (40% lumen stenosis), and severe stenosis in the right coronary artery (RCA) (80% lumen stenosis). The left ventriculogram showed important contrast extravasation from the left ventricle to the pericardium, with a giant pseudoaneurysm (Figure 2). A contrasted chest CT scan revealed cardiac enlargement with moderate to severe pericardial effusion and a discontinuity area in the left ventricle posterior-lateral wall communicating with the pericardial sac measuring 6.2 cm in its largest transverse diameter (Figure 3).

**Figure 4:**
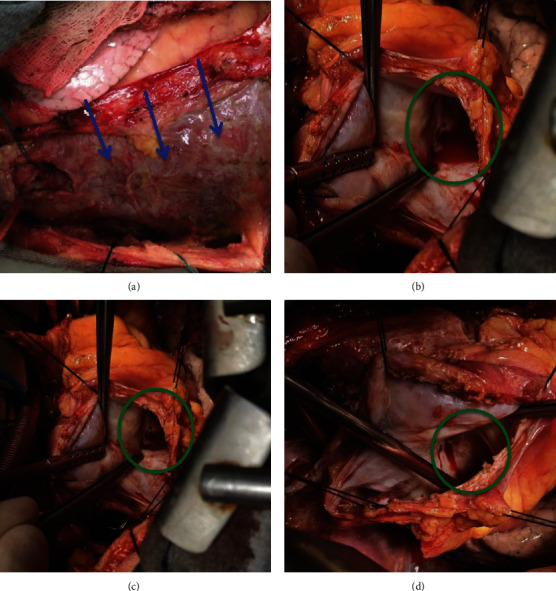
Intraoperative view. (a) Severe thickening of the pericardium with adherence of its layers (blue arrows), as commonly seen in constrictive pericarditis. (b–d) Rupture of the left ventricle (green circles) communicating the chamber and the pericardial sac.

**Figure 5:**
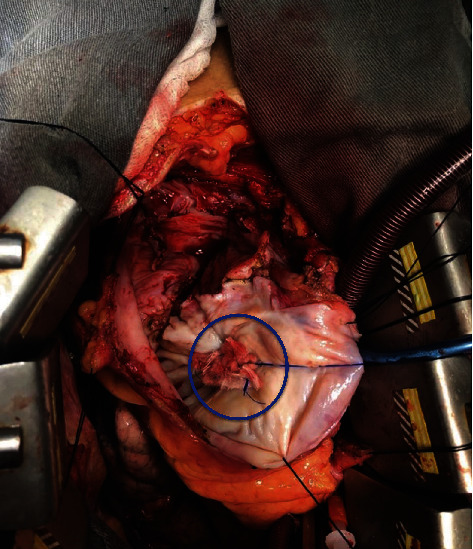
Intraoperative view. Suture of the myocardial rupture and pseudoaneurysm using linear teflon tissue (blue circle).

Surgical treatment was then performed through transsternal thoracotomy. Inspection of the mediastinum revealed severe thickening of the pericardium with adherence of its layers, suggesting constrictive pericarditis (Figure 4(a)). An attempt of dissection was unsuccessful. Transpleural cardiopulmonary bypass was initiated from the aorta to the right atrium, and transpleural access to the LVPA was obtained. Aortic clamping and cardioplegia were performed. Rupture of the left ventricle was noted (Figures 4(b)–4(d)), with fibrous tissue forming three distinct cavities inside the LVPA. Suture of the myocardial lesion and pseudoaneurysm using linear teflon tissue was performed (Figure 5), and anterior pericardiectomy was necessary because of severe pericardial thickening. At last, a bypass saphenous vein graft to the RCA was attempted, but it was impaired due to hostile pericardium anatomy caused by the LVPA.

The patient recovered well after the operation and was discharged from the hospital eight days later. At one-year follow-up, he was asymptomatic, with no signs of heart failure or complications. An echocardiogram showed mild diastolic dysfunction of the left ventricle, with anchoring patch in the lateral-apical wall and no signs of cavitary shunt to the pericardium. Ejection fraction was 72% by the Teichholz formula, and systolic and diastolic left ventricle measurements were, respectively, 32 and 56  mm. A control chest angio-CT scan was considered; however, with a good clinical outcome and serum creatinine persistently between 1.6 and 1.9 mg/dL, the exam was withdrawn. Percutaneous coronary intervention to RCA was deferred for the same reasons added to the lack of symptoms.

## 3. Discussion

In our case, the patient presented a giant chronic LVPA with no clear history of AMI. His symptoms were unspecific, and the diagnosis occurred accidentally after cardiac enlargement was seen in an abdominal CT scan. The rupture of the left ventricular myocardial wall favors ischemic myocardial lesion as etiology. The severe stenosis found in RCA may corroborate this hypothesis. However, diagnosis of the etiology of the LVPA was not clearly determined. Other possible causes previously described are surgical manipulation, penetrating or blunt trauma, and endocarditis with abscess. Due to the imminent risk of complete rupture, cardiac tamponade, and death, we decided for surgical treatment after a thorough heart team discussion.

The diagnosis of LVPA may have no specific symptoms or clinical signs. Chest pain, dyspnoea, or hypotension are common [9]. Pericardial friction and new cardiac murmurs are also possible [10], and sinus bradycardia or junctional rhythm has been reported in LVPA [11]. Most patients with LVPA present recurrent chest pain and signs of heart failure and as much as 10% present cardiac rhythm disturbances and syncope. However, 10% of the patients may be asymptomatic [11]. In the present case, the patient had mostly abdominal symptoms and also syncopes in two episodes.

Initial evaluation is usually done with an echocardiogram. After the suspicion, other imaging modalities offer better diagnostic accuracy, such as cardiac magnetic resonance, angio-CT, or cardiac catheterization [2]. In a case series, Yeo et al. showed that 48% of LVPA cases were incidentally diagnosed, mostly after imaging complementary exams [12]. In our case, the diagnosis of AMI was excluded, and imaging modalities incidentally showed cardiac abnormalities. Cardiac enlargement was found in abdominal CT scan, and then, as initial investigation targeted to heart failure, an echocardiogram revealed LVPA. After cardiac catheterization, the patient underwent a chest CT scan that provided detailed anatomic features of the LVPA.

The normal ejection fraction was calculated on an echocardiogram via the Teichholz formula, although with exclusion of the pseudoaneurysm. Quantification of systolic function including the area of the pseudoaneurysm could add a surgical value, aiding the prediction of improvement of the ejection fraction. Nevertheless, cardiac magnetic resonance (CMR) might be an important complementary imaging modality, with higher accuracy due to its higher spatial resolution [13]. CMR can distinguish a true left ventricle aneurysm from an LVPA by the finding of late enhancement predominantly in the myocardium or in the pericardium. Besides, CMR imaging is the ideal modality to perform LV function and adds important information on anatomic features of the LVPA [14, 15].

In the 1980s, the first surgical cases of LVPA correction used teflon patches for tamponade of ruptured areas [16]. In 1993, Padró et al. had 100% survival in 13 patients with LVPA after using biological tissue adhesive, with a median follow-up of 26 months [17]. In 2000, Pretre et al. showed better outcomes of surgical treatment over medical expectancy in patients with expanding LVPA, in both acute and chronic cases [18]. However, successful medical conservative treatment has been reported in isolated cases of LVPA [19, 20]. It is still unknown whether a small LVPA is less likely to rupture or which features represent greater risk of rupture and severe complications. Regarding its high surgical mortality, medical treatment may be considered for LVPAs less than 3 mm in size, aimed at decreasing pseudoaneurysm enlargement [14, 21]. Besides, postinfarction LVPAs located on the posteroinferior wall have the anatomical support of the diaphragm, facilitating the restraint of the ventricular cavity by the adjacent pericardium and making it more rupture-resistant [22]. Those favorable features implying stability are common in LVPAs that infrequently are not diagnosed and remain with conservative approaches.

Rupture of an LVPA may occur in 30 to 45% of all cases and is usually dramatic. Thus, the main objective of the surgical approach is to avoid progressive expansion and rupture of an LVPA [23]. Since it is a rare condition, there is a paucity of evidence on guiding therapeutic decisions for LVPA, and most available studies are case reports and case series. Comprehension of the etiology of LVPA is useful to define the best surgical strategy. Moreover, we propose an individual approach and shared understanding for decision-making. Heart team discussion is strongly recommended, including careful investigation on the etiology of LVPA in this process.

## Data Availability

The datasets used and analyzed during the current study are available from the corresponding author on reasonable request.
